# Treatment of reducible unstable fractures of the distal radius in adults: a randomised controlled trial of De Palma percutaneous pinning versus bridging external fixation

**DOI:** 10.1186/1471-2474-11-137

**Published:** 2010-06-29

**Authors:** João C Belloti, Marcel JS Tamaoki, Alvaro N Atallah, Walter M Albertoni, João BG dos Santos, Flavio Faloppa

**Affiliations:** 1 Departamento de Ortopedia e Traumatologia, Universidade Federal de São Paulo - Escola Paulista de Medicina, (Rua Borges Lagoa 786, 5° andar), São Paulo, (04038-032), Brazil

## Abstract

**Background:**

At present, there is no conclusive evidence regarding the best treatment method for reducible unstable fractures of the distal radius. This study compared the effectiveness of two methods used in surgical treatment of such fractures: percutaneous pinning and external fixation.

**Methods:**

We randomly allocated 100 patients into two groups treated surgically with modified De Palma percutaneous pinning and bridging external fixation. Independent but not blinded evaluators administered the DASH quality-of-life questionnaire at postoperative months 6 and 24, performed functional assessment of pain, range of motion, and palm grip strength, and radiographic examinations (volar and radial angle, and height of the radius) before the operation, immediately afterwards, and at 6 and 24 months postoperative. Modified De Palma percutaneous pinning patients used an above-elbow cast whereas external fixation group had unrestricted elbow motion after surgery. Patients who for any reason demonstrated treatment failure or required additional interventions were followed up and their results were included in the group into which these patients had initially been randomised according to the intention-to-treat principle. A significance level of 5% (alpha = 0.05). was used for all statistical tests, such that tests presenting a p-value less than 0.05 were considered statistically significant.

**Results:**

Ninety one (58.8 mean age and 66 participants were female) were included in the final assessment at 24 months. The DASH questionnaire evaluation showed a statistically significant result favouring the De Palma group (mean difference = -7.1 p = 0.044) after six months, but this was not maintained at 24 months. There were no statistically differences between the groups with respect to palm grip strength. Analysis of the range-of-motion limitation index (uninjured side minus affected side motion of) showed a statistical difference (mean difference = 2.4 p = 0.043) favoring the external fixator group with regard to the supination movement 6 months after the operation; however, this was not maintained at 24 months. The final results of the radiographic evaluation were similar for the two groups. Overall, five patients developed complications: two with De Palma pinning and three with external fixation.

**Conclusion:**

There was a small statistically significant difference favouring the De Palma method in early functional at 6 months according to the DASH questionnaire, and for supination movement favouring the fixator group. However, both were not clinical relevant. By 24 months the groups were similar for all outcomes

**Trial registration:**

Current Controlled Trials ISRCTN04892785

## Background

At present, there is no conclusive evidence regarding the best method of osteosynthesis for treatment of reducible displacement fractures of the distal radius that are potentially unstable [1].

External fixation and percutaneous pinning have both been described as good options for treating this type of fracture [1-3].

Several studies have described a variety of techniques using external fixators, but have not defined which one is most effective [2]. In the present study, we used bridging external fixation, since this method makes it possible to treat fractures with or without joint involvement. The De Palma method, originally described in 1952, used a single threaded pin through the ulna for fixation of fractures of the radius [3]. It has not had the same level of use as other methods of percutaneous pinning; nevertheless, some authors have described good results with a modified version of this method involving several Kirschner wires [4-6]. This encouraged us to adopt this approach for our study.

Comparative studies in the literature have not demonstrated any conclusive evidence regarding the superiority of percutaneous pinning compared with external fixation [1,7-9]. The aim of this study was to test the hypothesis that the modified De Palma method for percutaneous pinning would produce anatomical and functional results similar to those of bridging external fixation in the treatment of unstable reducible intra or extra-articular fractures of the distal radius in adult patients. Objective and subjective functional assessment, radiographic evaluation, complications and failures of the methods were considered in the final evaluation of the results.

## Methods

### Patient population: inclusion criteria

This study was designed in March 2002 and patients were recruitment between August 2002 and June 2004, with the last assessment in June 2006. The study protocol was approved by the university's ethics committee on August 9, 2002, under protocol number 0582/02.

The patients were adults aged over 40 years who presented with acute fractures with displacement up to 10 days old without previous treatment. The fractures were categorized using the Universal classification as unstable and reducible: type IIb (non articular) and type IVb (articular) [10]. Fractures were considered unstable if they presented three or more of the following factors at the initial radiographic examination: shortening of the radius by more than 5 mm; dorsal angulation greater than 20 degrees; joint incongruence; fracture associated with the styloid process of the ulna; dorsal comminution of the metaphysis; patient age greater than 60 years.

The criterion of fracture reducibility was verified using control radiographs in frontal and lateral views after anesthetizing the region and performing closed reduction. Fractures were considered reducible and eligible for inclusion in the study if they presented the following radiographic characteristics after reduction: shortening of the radius by less than 3 mm, joint fragment with displacement less than 2 mm, and dorsal displacement less than 10 degrees.

Patients were excluded if they presented fractures with volar angulation (Smith fracture), joint margin fractures (Barton fracture), open or bilateral fractures, or fractures that could not be reduced. We also excluded patients with previous histories of degenerative disease, wrist joint trauma, or traumatic injuries associated with the fracture that would make it impossible to apply the proposed surgical methods or evaluate the results. Furthermore, patients who refused to sign the free and informed consent statement were excluded.

### Study Design and Analysis

The patients were consecutively allocated to one of the two proposed treatment methods: modified De Palma transulnar percutaneous pinning or trans-articular linear external fixation. Allocation was performed according to instructions contained in 100 sealed opaque envelopes that had been sequentially numbered according to computer-generated randomisation. An independent person opened the envelopes during surgery.

This study was performed at Hospital São Paulo, Brazil, Universidade Federal de São Paulo, during 2002 to 2006. There was independent assessment of the DASH questionnaire, functional and radiographs outcomes. All assessors were not blinded.

The sample size was calculated beforehand, taking a confidence interval of 95%, statistical power of 90%, standard deviation of 15% in the DASH scores, and an absolute difference of 10% on DASH scores between Pinning and External Fixator. It was calculated that 47 patients were needed in each group. Allowing for a 6% loss to follow-up at 24 months, we aimed to recruit 50 patients into each group.

The Pearson chi-squared test was used to analyze the results from the two groups in relation to the categorical variables, and the Student's t test was used to compare the groups in relation to the numerical variables. Student's t test (parametric) was used to compare the clinical evolution of each group before the operation, just after the operation, and 6 and 24 months after the operation. A significance level of 5% (alpha = 0.05) was used for all statistical tests, such that tests presenting a p-value less than 0.05 were considered statistically significant.

Patients who for any reason demonstrated treatment failure or required additional interventions were followed up and their results were included in the group into which these patients had initially been randomised according to the intention-to-treat principle.

### Surgical intervention method

All patients were treated on an outpatient basis with reduction of fractures by manipulation with traction and counter-traction under anaesthesia by blockage of the brachial plexus or under general anaesthesia. Four previously designated surgeons with proven familiarity with both surgical techniques took part in the study. The surgical instruments needed for application of both treatment techniques were always available in the surgical room used for each operation. The technique to be used for each patient was only revealed intraoperatively, after radiological verification of fracture reducibility. At that time, the opaque sealed envelope was opened by independent person to reveal the treatment technique to be used. None of the patients underwent any specific treatment for associated fractures of the ulnar styloid.

### Surgical techniques

#### Modified De Palma technique of percutaneous pinning

**Figure 1:**
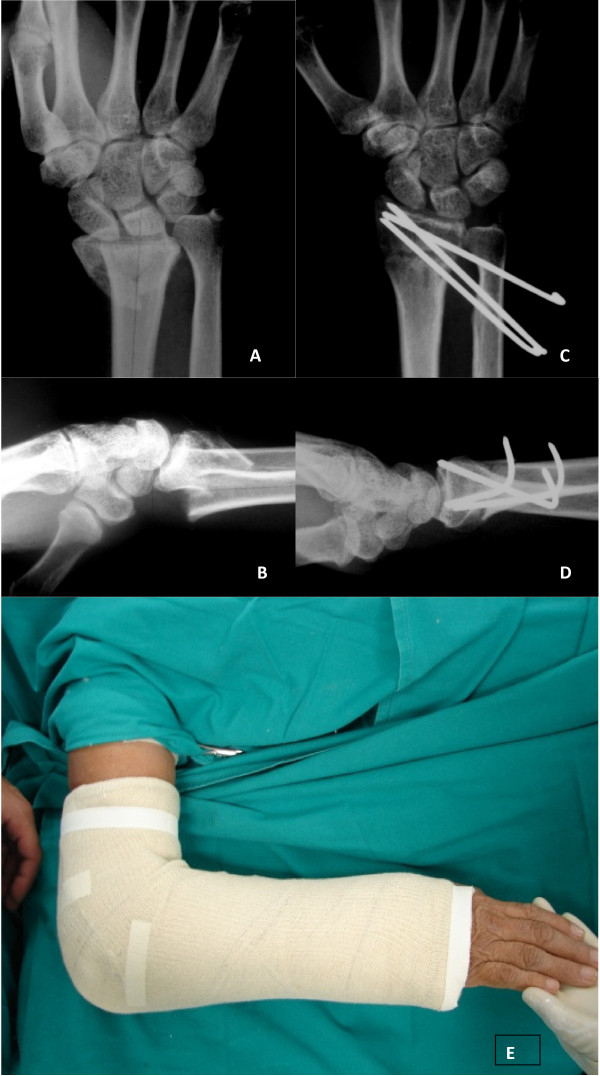
**Modified De Palma technique of percutaneous pinning**. **A**. Patient n° 62. Female, 58 years old. Radiograph preoperative in front view. **B**. Lateral view. **C**. The fracture of the radius is fixed using the ulna as a support. In the coronal plane we directed the convergent pins towards the styloid process of the radius. **D**. In the sagittal plane we directed the pin in diverging directions(dorsal and volar). **E**. Above elbow cast applied post-operative.

The modified De Palma fixation technique of percutaneous pinning was used in which the fracture of the radius was fixed using the ulna as a support [6]. Two to four Kirschner wires (1.5 to 2.0 mm) were introduced with the aid of fluoroscopy by means of stab incisions on the ulnar face of the distal region of the forearm, 2 to 4 cm proximal to the ulnar styloid process. In piercing through the two cortical walls of the ulna, we directed the convergent pins towards the styloid process of the radius in the coronal plane (figure 1C and 1D), with diverging directions (dorsal and volar) in the sagittal plane. When more than two pins were used for fixation of joint fragments, they were introduced tangentially to the joint surface of the radius (figure 1D). The pins were curved and cut close to the skin, and were protected with a bandage containing sterilized gauze. We then applied a cast that was extended above the elbow at 90 degrees with the forearm and wrist in neutral position (figure 1E). During postoperative follow-up, evaluations and rebandaging were performed every week. The cast and pins were retained for four to eight weeks, and the decision on when to remove them was based on radiographically demonstrated fracture consolidation. (Figure 1)

#### Bridging external fixation (Figures 2A, 2B and 2C)

**Figure 2:**
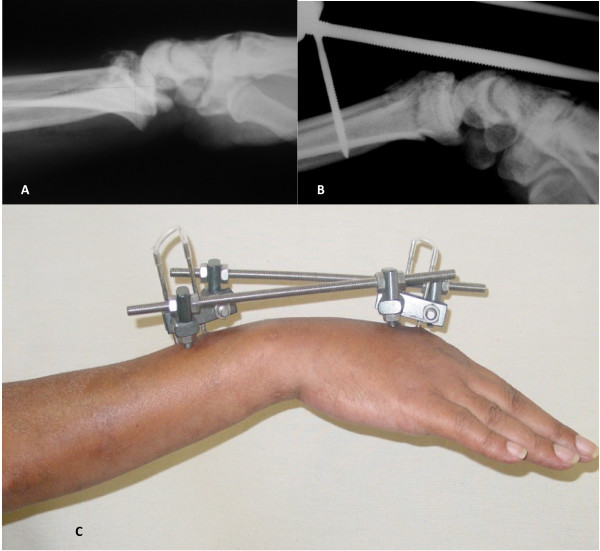
**Bridging external fixation technique**. **A**. Female, 50 years old. Radiograph preoperative in lateral view. **B and 2C**. The external fixator was positioned at an angle of approximately 90 degrees to the coronal plane of the forearm.

We used a bridging external linear fixator (Biomecanica^®^). Its placement started with the installation of two proximal pins. Longitudinal incisions of around 1 cm were made and the protective soft-tissue guide was introduced by means of blunt dissection. This was positioned at an angle of approximately 90 degrees to the coronal plane of the forearm. We then introduced two self-tapping 3.5 mm pins until they had pierced the volar cortical wall of the radius.

To place pins distally to the fracture, two 1 cm incisions were made in the dorsal face of the diaphysis of the second metacarpal bone. Then, with blunt dissection and the aid of a protective soft-tissue guide positioned at 90 degrees to the coronal plane of the hand, two 2.5 mm pins were introduced until they pierced the volar cortical wall of the second metacarpal. Following this, the site was bandaged with sterilized gauze and the patient was instructed to perform asepsis every day at the pin insertion sites using 2% chlorhexidine solution. There was no immobilization after this procedure and the external fixator was removed after six weeks. (Figure 2)

#### Rehabilitation

After surgery, all patients received the same instructions regarding general care and were assessed every week until fracture consolidation had been achieved. The group of patients that received the external fixator remained without any type of immobilization from the immediate postoperative period onwards. This allowed early mobilization of the distal radioulnar and elbow joints. After removal of the implants, all patients were kept without immobilization and underwent the same rehabilitation program, which included analgesia and gradually started passive and active mobilisation of wrist; followed by exercises for strength gain.

### Evaluation of the variables

#### Primary variables

All study participants were evaluated at 6 and 24 months after surgery. The assessor outcomes asked them to fill the DASH questionnaire, which has been validated for the Portuguese language [11]. The final score was calculated and transformed into the percentage functional limitation of the limb, using the specified weighting formula: final score = [{sum of the n responses/n} -1] × 25, where n is the number of completed responses. The two optional modules of the DASH questionnaire were not applied in this study.

To assess pain in the affected wrist, the assessor outcomes asked to participants to use a visual analog scale (VAS) in which pain level was expressed as an absolute value [12].

Radiographic evaluation was performed by two orthopaedists who were not directly associated with the study. The radiographs were measured as described by Kreder [13]. Radiographs in posteroanterior and lateral views were used for evaluation, and the following parameters were measured: angle, and a presence of a stepped joint. These measurements were made before the operation, immediately after the operation, and 6 and 24 months after the operation.

Fracture consolidation was defined as obliteration of the fracture lines and formation of bone callus, as observed on radiographs with frontal and lateral views.

#### Objective functional assessment

At 6 and 24 months after the operation, all patients underwent bilateral objective functional assessment consisting of goniometry and dynamometry by two independent physiotherapists. In the goniometric evaluation, the pronation-supination of the forearm, flexion-extension of the wrist and ulna, and radial deviation of the wrist were measured. Wrist grip strength was assessed using the Jamar^® ^dynamometer. The results were expressed as the difference in values between the uninjured and affected sides (index of limitation).

#### Complications and failures

'All adverse events which resulted in patients requiring additional clinical treatment outside of standard care were considered to be complications. It was deemed that the surgical method had failed if the patient required a new surgical procedure or the initially allocated treatment had to be halted.

The functional and radiographic evaluations, pain measurements using the VAS, and applications of the DASH questionnaire were performed by professional orthopaedists and physiotherapists who were not directly associated with the study.

## Results

All patients initially attended the orthopaedics emergency clinic of our hospital. The surgical interventions and postoperative follow-up over a minimum of 24 months also took place at this hospital.

**Figure 3:**
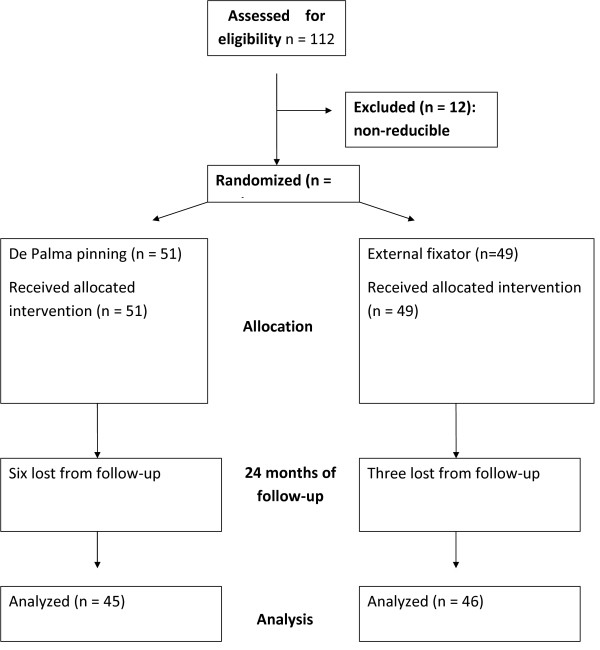
**Flow of participants**. Diagram showing the flow of participants through each stage of the study.

Out of the 100 patients who underwent the surgical intervention, 91 were included in the final assessment at 24 months (91%). Of the nine patients who did not undergo the final assessment, one died (external fixator Group) and eight abandoned the study and could not be located due to address changes despite persistent attempts to locate them (figure 3). Clinical and demographics are shown at table 1.

**Table 1:** Demographic characteristics of the two patient groups

Characteristic	De Palma pinning group (n = 51)	External fixator group (n = 49)	p
Mean age (standard deviation)	57.5 (11.9)	59.2 (12.7)	0.501
Sex (female)	39	34	
Side affected (right)	27	23	0.548
Handedness (right)	48	46	0.960

### Main outcomes

#### DASH questionnaire and pain evaluation

Patients who underwent the modified De Palma technique for percutaneous pinning presented better results after six and 24 months with regard to functional limitation (DASH), compared with patients treated with the external fixator. However, this finding only reached statistical significance for evaluation using the DASH questionnaire after six months, of follow-up (mean difference = -7.1 p = 0.044) (Table 2). There were no statistical differences between two groups when pain scores (VAS) were assessed. (Table 2).

**Table 2:** Main outcomes

Outcome	DePalma Pinning Mean (SD)	External Fixator Mean (SD)	P value
**Six months**			
**N**	48	48	
**DASH**	16.5 (14.7)	23.6 ( 18.1)	0.044
**Pain (cm)**	2.8 (1.8)	3.5 (2.0)	0.08
**24 months**			
**N**	45	46	
**DASH**	9.4 (12.9)	12.9 (15.2)	*0.24*
**Pain (cm)**	1.2 (1.4)	1.4 (1.5)	*0.59*

N - Number of patients

DASH - percentage values for limb limitation: low values indicate less limitation.

Pain (VAS) - absolute values (cm): low values indicate low pain.

0: no pain to 10 cm: worst pain imaginable

### Secondary outcomes

#### Grip strength

Comparative analysis of the grip strength limitation index (uninjured side minus affected side grip strength) showed similar results for the two groups at both 6 and 24 months after surgery (Table 3).

**Table 3:** Secondary outcomes

Outcome	DePalma Pinning Mean (SD)	External Fixator Mean (SD)	P value
**Six months**			
**N**	48	48	
**Grip (Kgf)**	5.6 (8.2)	5.8 (8.9)	0.89
*Flexion	16.7 (12.5)	20.4 (11.9)	0.14
*Extension	17.0 (11.5)	21.3 (15.2)	0.13
*Ulnar desviation	8.2 (7.8)	7.8 (7.5)	0.82
*Radialdesviation	4.6 (4.4)	6.0 (5.1)	0.18
*Pronation	15.7 (11.7)	16.8 (12.2)	0.64
*Supination	23.4 (16.7)	16.6 (14.3)	0.043
**24 months**			
**N**	45	46	
**Grip (Kgf)**	3.1 (8.0)	2.2 (6.6)	*0.55*
*Flexion	7.0 (12.1)	6.0 (8.6)	*0.63*
*Extension	5.3 (9.4)	6.4 (9.3)	*0.59*
*Ulnar desviation	2.8 (5.0)	4.2 (5.9)	*0.22*
*RadialDesviation	21 (4.0)	1.6 (2.6)	*0.52*
*Pronation	4.2 (8.9)	5.0 (7.3)	*0.60*
*Supination	5.8 (10.4)	5.4 (10.9)	*0.84*

N - Number of patients

Mean limitation in degrees (control side minus affected side)

P value - 95% confidence interval

* Units of measurement = degrees

#### Range of motion

Analysis of the range-of-motion limitation index showed a statistical difference (p = 0.043) favouring the external fixator group with regard to the supination movement 6 months after the operation; however, this was not maintained at 24 months. For all other measurements, the results were similar between the groups (Table 3).

### Radiographic results

Preoperative radiographic data showed that the distribution of fracture severity was similar between the two treatment groups. Both surgical techniques showed good radiographic results immediately after the operation. Overall, 16 patients presented with articular incongruence (gap or step off) in the final assessment: seven in the external fixator group (five with 1 mm, one with 1.5 mm and one with 2 mm) and eight in De Palma group (five with 1 mm, two with 2 mm and one with 3 mm), with no statistically significant difference (p = 0,549).

We observed progressive loss of the initial reduction in both treatment groups; however, only the radial inclination showed a statistically significant loss favoring the De Palma technique at the 24-month assessment. In the final evaluation, both techniques were shown to be effective for correcting the initial deformity (table 4).

**Table 4:** Radiographic results

Outcome	Technique	Mean	Standard Deviation	P
Volar angle before operation	De Palma	-22.8222	9.9414	0.406
	External fixator	-24.7609	12.1146	

Volar angle just after operation	De Palma	8.4000	4.3453	0.075
	External fixator	6.6522	4.9000	

Volar angle 6 months	De Palma	7.4444	5.8758	0.057
	External fixator	4.8696	6.8203	

Volar angle 24 months	De Palma	6.8667	6.6250	0.094
	External fixator	4.4000	7.1903	

Radial angle before operation	De Palma	11.4444	6.3445	0.294
	External fixator	12.6522	4.3626	

Radial angle just after operation	De Palma	21.7333	2.2603	0.090
	External fixator	20.8478	2.6580	

Radial angle 6 months	De Palma	20.7111	3.1811	0.120
	External fixator	19.6739	3.1273	

Radial angle 24 months	De Palma	20.4444	3.5964	0.049
	External fixator	18.8222	4.0915	

Height of radius before operation	De Palma	2.7778	3.5985	0.240
	External fixator	1.9130	3.3654	

Height of radius just after operation	De Palma	9.9556	2.2859	0.207
	External fixator	9.3043	2.5980	

Height of radius 6 months	De Palma	8.8889	2.8382	0.180
	External fixator	8.0435	3.1266	

Height of radius 24 months	De Palma	8.4000	3.4005	0.388
	External fixator	7.7778	3.4105	

Units of measurement = degrees

### Complications and failures

Overall, five patients developed complications: two with De Palma pinning and three with external fixation. Of these, two were considered failures of the method (one with De Palma pinning and one with external fixation). In the De Palma technique group, one patient suffered a fracture of the ulna after the synthesis material had been removed and was treated conservatively with cast. A second patient developed a deep infection that required surgical cleaning and modification of the surgical method; this case was considered a failure of the method.

In the external fixator group, two patients developed deep infection that was treated conservatively with antibiotic therapy. A third patient had pseudoarthrosis of the fracture of the distal radius that required other surgical procedures and changes in the treatment method (dorsal plate and iliac bone graft); this case was considered failure of the method.

## Discussion

A literature search failed to provide sufficient data to determine the best form of treatment for fractures of the distal radius, particularly with regard to potentially unstable fractures with or without joint involvement [1,14]. However, there were trends favouring the use of percutaneous pinning and external fixators therefore in this study we compared these two methods [1]. Since we did not find any evidence favouring a specific technique for pinning or external fixation, we chose to compare the modified De Palma method and the bridging external fixation method [2,6,15]. Both of these methods are used in our country and are readily accessible methods for treatment of this type of fracture [16].

The method originally described by De Palma used a single threaded wire and may not provide sufficient stability for unstable comminutive joint fractures [3]. Thus, there was an initial limitation on the use of this technique, although Dowling & Sawyer performed operations on 50 patients using this method and obtained satisfactory results for extra- and intra-articular fractures [17]. The fixation principle originally used by De Palma was subsequently modified by Rayhack who described a transulnar pinning method using several Kirschner wires, and by Toledo who used two to four Kirschner wires [4,6]. Both of these modifications have demonstrated good functional and radiographic results with low complication rates, and this encouraged us to use this method for percutaneous pinning.

Overall analysis of our results showed that the De Palma percutaneous pinning method was as effective as the external fixation method when analyzed after 24 months of follow-up. In the literature, three other prospective studies have compared other percutaneous fixation methods with external fixation and similarly did not find any statistically significant difference between the two fixation methods [7-9].

In analyzing our results from the DASH questionnaire, we found a statistically significant difference favoring the modified De Palma method at six months that was not sustained in the final evaluation at 24 months; therefore, this difference at six months (7.1 points) was not clinically relevant. We believe that this initial difference reflects the lower pain index in the De Palma group compared with the external fixator group, although this did not reach statistical significance. There are no comparable reports in the literature, since earlier studies that compared the same fixation methods used other methods of evaluation.

With respect to the range of motion, the two groups were found to be similar except for a significant difference favoring the external fixation method in the supination movement after six months. This was probably caused by blockage of the distal radioulnar joint and the use of above elbow cast in the De Palma technique. This difference was not maintained in the 24-month evaluation. Other studies are consistent with our findings, except for that of Franck which demonstrated a statistically significant difference in range of motion favouring patients treated with an external fixator [7-9]. However, these results may have been influenced by the fact that the final evaluation was performed after six months, only extra-articular fractures were treated, and a non-bridging external fixator was used.

Our radiographic results indicated a trend favoring percutaneous pinning: there was a statistically significant difference favoring the De Palma method with regard to loss of radial inclination evaluated after 24 months. However, this difference is not clinical relevance.

The two treatment techniques had similar results with respect to complications and treatment failures. We did not observe any reflex sympathetic dystrophy or lesions of the sensitive branches of the radial nerve that would have required additional therapeutic measures in either of the treatment groups. We only observed transitory states of pain and edema that did not require additional intervention and therefore were not classified as complications. This result contrasts with the literature: Ludvigsen found 13 cases of lesions of the superficial radial nerve, of which five had persistent symptoms [8]. We believe that the divergence between the results from our study and those of other authors results from both the technique we used to apply the external fixator (with the introduction of pins in the dorsal region of the forearm) and the use of De Palma transulnar percutaneous pinning (which avoided approaching the superficial radial nerve and its branches). Future clinical studies comparing the techniques for placing the pins for the external fixator (dorsal or dorsal-radial), and comparing transulnar percutaneous pinning with pinning techniques that use accesses in the region of the superficial radial nerve and its branches, are required to test this hypothesis.

Our study had some limitations. Firstly, the strength of our results was limited by small sample size, because we would have needed to treat approximately 600 patients to reach an ideal statistical power, which would have required a multicenter study. Moreover, when we designed our study protocol we adopted epidemiological and radiographic criteria for including patients with unstable fractures although we did not find any conclusive evidence in the literature that would safely allow predictions regarding the instability of the fracture based on initial radiographic examination [18]. However, after beginning our study, we found new evidence relating to the better prediction of instability, which can be used to inform future research [19]. Even so, our results demonstrated that patients with characteristically unstable fractures were included in this study. Finally, because of the different access routes and fixation methods used in these techniques, the evaluators of the radiographic and functional results could not be blinded. Thus, to minimize any biasing, evaluations were performed by professionals who were not associated with the study.

Our study demonstrated that although the modified De Palma transulnar percutaneous pinning method is not widely covered in the literature, when compared with external fixation it proved effective for treating unstable intra or extra-articular fractures in adult patients. Other authors have similarly described good clinical results from this percutaneous pinning method [4-6]. Moreover, Franck reported that the purchase cost of the external fixator was approximately nine times greater than that of percutaneous pins [7]. A similar cost difference exists in our country.

Considering that the success and complication rates in the two treatment groups were similar, it can be concluded that this pinning technique provides a good alternative approach for treating reducible unstable fractures of the distal radius. However the pinning requires elbow immobilization that might be a source of early impairment in early the range of motion.

## Conclusion

There was a small statistical significant difference favouring the De Palma method in functional early analysis (6 months) according to the DASH questionnaire, and for supination movement favouring the fixator group. However, both were not clinical relevant. At 24 months, the groups were similar for both groups.

## Competing interests

The authors declare that they have no competing interests.

## Authors' contributions

JCB participated in the design of the study, revised the manuscript, provided clinical care to participants during the follow-up, collected data and the bibliography for references. MJST conceived the study design, treated patients surgically and revised the manuscript. JBGS treated patients surgically, provided clinical care to participants during the follow-up and translate the manuscript to English. AA participated in design of the study, revised the draft and manuscript and performed statistical analysis. FF participated in design of study, treated patients surgically and transcribed the data for statistical analysis. WMA revised bibliography for references, the article and gave the final approval of the version to be published. All authors read and approved the final manuscript.

## Pre-publication history

The pre-publication history for this paper can be accessed here:

http://www.biomedcentral.com/1471-2474/11/137/prepub
